# Genetic Diversity and Detection of Respiratory Viruses Excluding SARS-CoV-2 during the COVID-19 Pandemic in Gabon, 2020–2021

**DOI:** 10.3390/v16050698

**Published:** 2024-04-28

**Authors:** Georgelin Nguema Ondo, Yuri Ushijima, Haruka Abe, Saïdou Mahmoudou, Rodrigue Bikangui, Anne Marie Nkoma, Marien Juliet Veraldy Magossou Mbadinga, Ayong More, Maradona Daouda Agbanrin, Christelle M. Pemba, Romuald Beh Mba, Ayola Akim Adegnika, Bertrand Lell, Jiro Yasuda

**Affiliations:** 1 Centre de Recherches Médicales de Lambaréné, Lambaréné BP.242, Gabon; georgelinkluivert9@gmail.com (G.N.O.); saidou.mahmoudou@cermel.org (S.M.); bikrod16@gmail.com (R.B.); mymhankoma@yahoo.fr (A.M.N.); julietveraldy@yahoo.fr (M.J.V.M.M.); ayongterence@gmail.com (A.M.); daouda_agbanrin@yahoo.fr (M.D.A.); behmbaroms@gmail.com (R.B.M.); aadegnika@cermel.org (A.A.A.); bertrand.lell@cermel.org (B.L.); 2 Graduate School of Biomedical Sciences, Nagasaki University, Nagasaki 852-8523, Japan; 3 Department of Emerging Infectious Diseases, Institute of Tropical Medicine (NEKKEN), Nagasaki University, Nagasaki 852-8523, Japan; ushijima-yu@md.tsukuba.ac.jp (Y.U.); abeh@nagasaki-u.ac.jp (H.A.); 4 Division of Biomedical Science, Institute of Medicine, University of Tsukuba, Tsukuba 305-8577, Japan; 5 Vietnam Research Station, Institute of Tropical Medicine (NEKKEN), Nagasaki University, Nagasaki 852-8523, Japan; 6 Department of Emerging Infectious Diseases, National Research Center for the Control and Prevention of Infectious Diseases (CCPID), Nagasaki University, Nagasaki 852-8523, Japan; christellepemba@nagasaki-u.ac.jp; 7 Division of Infectious Diseases and Tropical Medicine, Medical University of Vienna, 1090 Vienna, Austria

**Keywords:** Gabon, respiratory viruses, genetic diversity, incidence, COVID-19 pandemic

## Abstract

Acute respiratory infections are a major global burden in resource-limited countries, including countries in Africa. Although COVID-19 has been well studied since the pandemic emerged in Gabon, Central Africa, less attention has been paid to other respiratory viral diseases, and very little data are available. Herein, we provide the first data on the genetic diversity and detection of 18 major respiratory viruses in Gabon during the COVID-19 pandemic. Of 582 nasopharyngeal swab specimens collected from March 2020 to July 2021, which were SARS-CoV-2 negative, 156 were positive (26%) for the following viruses: enterovirus (20.3%), human rhinovirus (HRV) (4.6%), human coronavirus OC43 (1.2%), human adenovirus (0.9%), human metapneumovirus (hMPV) (0.5%), influenza A virus (IAV) (0.3%), and human parainfluenza viruses (0.5%). To determine the genetic diversity and transmission route of the viruses, phylogenetic analyses were performed using genome sequences of the detected viruses. The IAV strain detected in this study was genetically similar to strains isolated in the USA, whereas the hMPV strain belonging to the A2b subtype formed a cluster with Kenyan strains. This study provides the first complete genomic sequences of HRV, IAV, and hMPV detected in Gabon, and provides insight into the circulation of respiratory viruses in the country.

## 1. Introduction

Acute respiratory infections (ARIs) are a major global burden, particularly in resource-limited countries, such as those in Africa, where ARIs are considered a major cause of morbidity and mortality, and where a higher number of influenza-associated hospitalizations have been reported [1]. Viral infections of the upper and lower respiratory tracts are among the most common diseases in humans, and are a major cause of morbidity and mortality worldwide [1]. Viral respiratory infections are the most common causes of pediatric diseases [2].

As represented by the novel coronavirus disease (COVID-19), emerging respiratory viruses can cause severe outbreaks with substantial morbidity and mortality [3]. Due to the extensive surveillance studies on COVID-19, other respiratory viral diseases have attracted less attention from researchers and public health organizations since the onset of the pandemic. However, the improvement of preventive sanitation (such as hand washing, the wearing of masks, and containment) induced by COVID-19 may have an impact on the incidence of ARIs caused by respiratory viruses [4,5]. Therefore, ARI surveillance during a pandemic is important for public health.

Respiratory infections are a major public health concern in sub-Saharan Africa, with a higher mortality rate than that of other regions [6]. Several studies have reported the circulation of enteroviruses (EVs) and human rhinoviruses (HRVs) in patients with mild-to-severe respiratory infections [7]. Studies have shown that the burden of respiratory syncytial virus (RSV) infection is high among children less than two years of age who show symptoms of ARI in Cameroon [8], and that ARIs are the most frequent cause of medical consultation in the Central African Republic [9]. Owing to the limited number of previous reports on the situation of ARIs in Central Africa, further surveillance of ARIs is required to investigate their circulation and causative agents in order to develop effective countermeasures against ARIs.

In Gabon, a Central African country, only one study reported the circulation of respiratory viruses in cases of influenza-like illnesses between 2010–2011 [10]. Although the data of this study provide a better understanding of the circulation of respiratory viruses in Gabon through the detection of EV, HRV, RSV, parainfluenza virus (PIV), and influenza viruses, this study did not include any sequence analysis. No surveillance studies have been conducted in the country since 2011, and little is known about the causative agents of ARIs in Gabon. Our study aims to investigate the situation of ARIs other than COVID-19 in Gabon during the COVID-19 pandemic, and to infer the phylogenetic relationships of the respiratory viruses detected in this study.

## 2. Materials and Methods

### 2.1. Ethics Statement

This study was approved by the Institutional Review Board of the Centre de Recherches Médicales de Lambaréné (CERMEL) and the National Ethical Committee in Gabon (approval numbers CEI-001 and PROT Nº 065/SG/P/CNER, respectively).

### 2.2. Sample Collection

**Figure 1:**
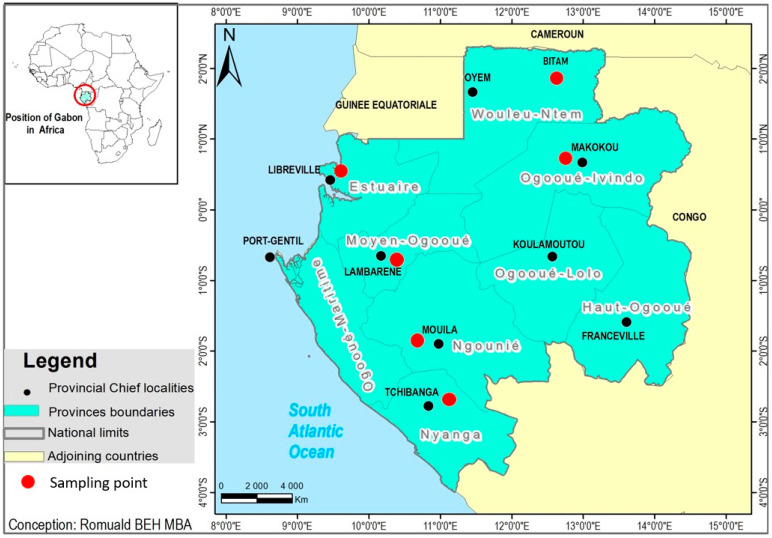
Location of the study area. Lambaréné (study site) and other major cities in Gabon. Black dots depict the state capital city of Gabon, and red dots depict the cities where the samples were collected.

The present study is a national cross-sectional study, as the samples were from several cities, and it was conducted in Gabon, Central Africa. From March 2020 to July 2021, nasopharyngeal swab specimens were collected for testing COVID-19 in suspected cases a follows: (A) the onset of COVID-19-like symptoms after staying in a community of the COVID-19 transmission area 14 days prior to the onset; (B) hospitalized individuals who were reported as COVID-19 suspected cases by physicians. Samples were kept in a swab containing a UTM-RT-mini transport medium (Copan Italia S. p. A., Brescia, Italy; Copan Diagnostics Inc. Jefferson Avenue, Murrieta, CA, USA) while shipping to CERMEL and then stored at −80 °C until analysis. All the samples were collected in the context of the response to the COVID-19 epidemic in Gabon, which explains why the number of samples was low and unequal. Samples were routinely tested for COVID-19 in CERMEL after viral RNA extraction. The present study used residual swab samples which were confirmed to be COVID-19 negative. The samples used in this study were collected from individuals aged >1 year in six cities, which were Gabon, Libreville, Lambaréné, Bitam, Mouila, Tchibanga, and Makokou. The sample collection points are shown in a map generated using ArcGIS software (Environmental Systems Research Institute, Inc., USA), and the map data were downloaded from diva-GIS (accessed on 10 May 2022, http://www.diva-gis.org/) (Figure 1). The demographic information of the samples was collected after full anonymization by the CERMEL diagnosis team.

### 2.3. Viral RNA Extraction and Detection of Respiratory Viruses Using Reverse Transcription-Quantitative PCR (RT-qPCR)

Viral RNA was extracted from 140 μL of the sample using a QIAamp Viral RNA Mini Kit (Qiagen, Hilden, Germany) according to the manufacturer’s instructions. RT-qPCR was performed in a 20 μL reaction using a QuantiTect Probe RT-PCR Kit (Qiagen) [11]. The PCR primer and probe sequences used for the detection of targets have been previously described as follows: human parechovirus [12], human metapneumovirus (hMPV) [13], human coronaviruses (hCOVs) (229E, NL63, OC43, and HKU1) [14], EV [15], human adenovirus (HAdV) [16], RSV [17], PIVs (types 1, 2, and 3) [18], PIV subtypes 4a and 4b [19], HRV [20], influenza A/B viruses (IAV/IBV) [21], and measles virus [22]. The final concentration of primers and probes was set to 1 μM and 0.1 μM, respectively. RT-qPCR assay was carried out using a StepOnePlus instrument (Thermo Fisher Scientific, Waltham, MA, USA) under the following conditions: 30 min at 50 °C, 15 min at 95 °C and 45 cycles of 15 s at 95 °C, and 60 s at 60 °C. Data collected from the RT-qPCR assays were analyzed using StepOne software v2.3, and samples showing threshold cycle (Ct) values < 40 were considered positive [23].

### 2.4. Viral Genome Sequencing and Genotype Determination

To determine HRV types, the amplification of the VP4/VP2 (541 nucleotides) region of the viral genome was performed for HRV-positive samples using the PrimeScript II High-Fidelity One Step RT-PCR Kit (Takara Bio, Shiga, Japan) with previously reported primers [24]. After agarose gel purification with the QIAquick Gel Extraction Kit (Qiagen), PCR products were processed with the BigDye Terminator v3.1 Cycle Sequencing Kit (Thermo Fisher Scientific) and analyzed using an ABI3500 capillary sequencer (Thermo Fisher Scientific) to obtain sequence data. Sequenced fragments were assembled manually using Bioedit software v7.2.5 (https://thalljiscience.github.io/). The genotypes of the detected strains were determined using BLAST (https://blast.ncbi.nlm.nih.gov/Blast.cgi). RT-PCR for EV- and HCoV OC43-positive samples or PCR- for HAdV-positive samples were performed using the following primers: EV [25], HCoV OC43 [26], and HAdV [27], although it was difficult to amplify the viral genomes. Regarding PIVs, we did not attempt PCR on positive samples because their Ct values were >38.

### 2.5. Whole-Genome Sequencing

The whole-genome sequencing of HRV, IAV, and hMPV was performed using samples with low Ct values (<35). RNA samples were randomly amplified using a REPLI g WTA Single Cell Kit (Qiagen, Hilden, Germany). Libraries were prepared using the NEBNext Ultra II FS DNA Library Prep Kit for Illumina (New England Biolabs, Ipswich, MA, USA) in combination with NEBNEXT Multiplex Oligos for Illumina (Dual Index Primers Set 1 and Set 2) (New England Biolabs) according to the manufacturer’s instructions. After the libraries were quantified using the NEBNext Library Quant Kit (New England Biolabs), sequencing was performed using the Miniseq High Output Kit on a Miniseq sequencer (Illumina, San Diego, CA, USA). The read data were trimmed and mapped to a reference sequence using CLC Genomics Workbench software (Qiagen). Consensus sequences were extracted as complete or nearly complete.

### 2.6. Phylogenetic Analysis

The genomic nucleotide sequences of the detected viruses were aligned with the reference sequences using MEGA 11.0.8 software with ClustalW [28,29]. Reference sequences for HRV and IAV were obtained from GenBank, whereas those for hMPV were obtained from the VIPR database. Multiple sequence alignments were processed using MAFFT, and phylogenetic trees were constructed using the maximum-likelihood method with 1000 ultrafast bootstraps in the MEGA software. Phylogenetic trees were visualized and modified using FigTree v1.4.4 (http://tree.bio.ed.ac.uk/software/figtree/) [30].

### 2.7. Statistical Analysis

Statistical analyses were performed using GraphPad Prism version 9 (GraphPad Software, Boston, MA, USA). Results were considered statistically significant when the *p*-value was less than 0.05.

### 2.8. Sequence Data Availability

The viral genome sequences obtained in this study have been deposited in GenBank under the following accession numbers: LC789193, LC789530–LC789532, and LC789925–LC789935 for HRV; LC789936 for hMPV; and LC790011–LC790018 for IAV.

## 3. Results

### 3.1. Sample Population

Between March 2020 and July 2021, 582 swab samples were collected from COVID-19-negative individuals who were presenting with respiratory symptoms in several regions of Gabon. The participants included 247 men (42.4%) and 268 women (46.1%); 67 (11.5%) were unspecified. The women-to-men ratio was 0.52, and the mean age was 38.5 years (1–80 years). All individuals were sampled only once, owing to their visiting sampling centers for COVID-19 testing. The sociodemographic characteristics of the study participants are summarized in Table 1. Adults were included in 81.1% of the participants, and 70.4% of the samples were collected from the capital city of Libreville. The geographic distribution of the sampling points was as follows: 410 samples (70.4%) from Libreville, 82 (14.1%) from Lambaréné, 48 (8.3%) from Bitam, 14 (2.4%) from Mouila, 9 (1.6%) from Tchibanga, and 5 (0.9%) from Makokou. The distribution of age was as follows: 15 participants (2.6%) were in the age group of 0 to 5 years old, 35 (6.1%) in 6–17 years old, 452 (77.7%) in 18–60 years old, and 20 (3.4%) in 60 years and older (Table 1).

### 3.2. Detection of Respiratory Viruses

A total of 582 samples were screened for 18 respiratory viruses as follows: EV, HRV, HAdV, hMPV, IAV, IBV, seasonal coronaviruses (229E, NL63, OC43, and HKU1 strains), PIV (types 1, 2, 3, 4a, and 4b), human parechovirus, RSV, and measles virus. A total of 154 samples (26.5%) tested positive for at least one virus (Table 1). The eight following viruses were detected: EV, HRV, HCoV-OC43, HAdV, hMPV, IAV, PIV-3, and PIV-4a. The overall prevalence of each detected virus in 582 individuals, regardless of demographic information, was as follows: EV, 20.3% (118/582); HRV, 4.6% (27/582); HCoV-OC43, 1.2% (7/582); HAdV, 0.9% (5/582); hMPV, 0.5% (3/582); IAV, 0.3% (2/582); PIV-4a, 0.3% (2/582); and PIV-3, 0.2% (1/582). EV was the most prevalent virus (20.3%), followed by HRV (4.6%) (Table 1). The positivity rate for viral infections was similar between men (26.3%) and women (26.9%) (*p* = 0.29). The positivity rate was 53.3% in the age group of 0 to 5 years, 25.7% in the 6–17 years, and 25.2% in the 18 year and older age group (*p* = 0.23). There were no significant differences in the positivity rates between sexes or age groups. Among the detected viruses, EV and HRV were prevalent in all study regions, whereas IAV was detected in specific cities (Table 1). This trend is consistent with a previous study conducted in Gabon between 2010 and 2011 [10]. In addition, a previous study showed that influenza was most frequently detected from November to December, whereas, in our case, influenza was detected in July.

### 3.3. Monthly Changes of Viral Infection and Coinfection Cases

To verify the most prevalent time points, monthly cases of viral infections were counted over the study period. The number of viral detections per month is shown in Table 2. April 2020 was the most prevalent month, with 88 positive samples, including 64 EV-positive samples. EV was detected in every sampling month, whereas HCoV-OC43, PIV-3, and PIV-4a were detected only in April 2020, and IAV was detected only in July 2021. Eleven cases of coinfection were identified, including EV/HRV, HAdV/hMPV, HAdV/PIV-4a, and EV/IAV (Table 2). Among the positive samples, single viral infections accounted for 92.8% (143/154) of the infection cases, whereas infections with multiple viruses were observed in 7.2% (11/154) (Table 2). Notably, 44 samples were examined between December 2020 and April 2021, and none were positive. In addition, no samples were examined in May or June 2021 because the participants did not meet all of the inclusion criteria (not cases at D0).

### 3.4. Circulation of Respiratory Viruses in Gabon

Samples were collected from six different cities—Libreville, Lambaréné, Bitam, Mouila, Tchibanga, and Makokou—in Gabon for the COVID-19 diagnostic test under the national protocol to prevent COVID-19 spread. To investigate the regional trend of respiratory viruses, the detection rates of the viruses were calculated in each province where the sampling was conducted. At least one virus was detected in all of the study areas. Tchibanga had the highest viral detection rate (55.6%), followed by Bitam (31.2%), Lambaréné (29.3%), and Libreville (25.9%) (Table 1).

### 3.5. Genotyping and Phylogenetic Analysis of Detected HRV Strains

To determine the genotypes of the viruses detected in this study, partial viral genomes were amplified using PCR. Despite attempts to obtain PCR products from EV-, HRV-, HCoV-OC43-, HAdV-, and PIV-positive samples, only 15 HRV genomic fragments were obtained, possibly because of the extremely low titers of viruses in most positive samples. BLAST analysis revealed that 53.3% (8/15) of the HRV strains belonged to species A, 26.7% (4/15) to species B, and 20.0% (3/15) to species C, including one sample that was coinfected with HRV-A and HRV-B (Table 3). 

**Figure 2:**
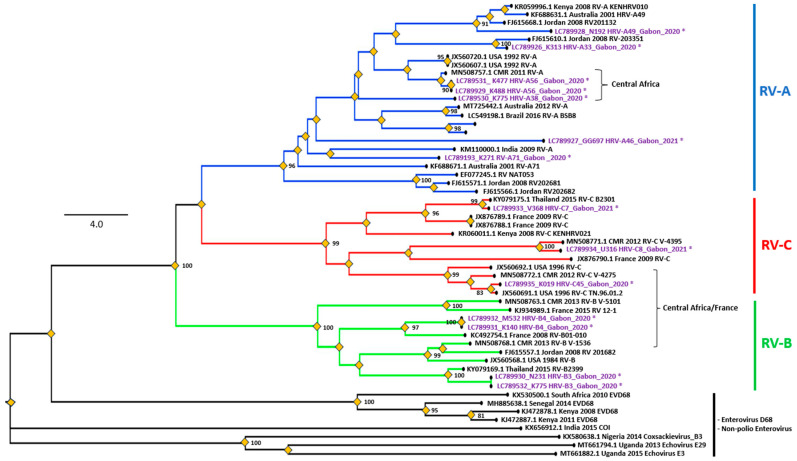
The rooted Phylogenetic tree of the human rhinovirus was generated based on the VP4/VP2 region. A maximum likelihood tree was inferred with 1000 bootstrap replicates; Enterovirus D68 and Non-polio Enterovirus were used as the outgroup to root the tree. The scale bars represent the frequency of nucleotide substitutions, and the numbers on the nodes of the branches are determined bootstrap values. Only values ≥ 70% are presented. The reference sequences of different continents, obtained on GenBank, are identified from the left to the right with the access number, the origin country, and the species attributed by the author. The strains identified in this study are colored purple with asterisks. Species are indicated on the right in colors as follows: A (blue), B (green), and C (red). Scale bar: Nucleotide substitutions per site.

To infer the phylogenetic relations of the HRV strains detected in this study, a phylogenetic analysis based on the VP4/VP2 region sequence was performed with the reference strains (Figure 2). Two HRV-A isolates in this study (K477 and K488) were genetically similar to the Cameroon-2011 strain (MN508757), which was detected in a country neighboring Gabon, and may indicate cross-border transmission. The HRV-A strains detected in this study were as follows: A56 (two strains), A22, A33, A49, A46, and A71 (one strain each). The types of HRV-B strains were as follows: B4 (two strains) and B3 (one strain). The HRV-C strains were as follows: C7, C8, and C42 (one strain each). The HRV-C8 and C42 strains were detected in Cameroon in 2021 by Kenmoe et al. [31]. This confirms the exchange of pathogen strains between two neighboring countries, Cameroon and Gabon, indicating the importance of cross-border surveillance.

**Figure 3:**
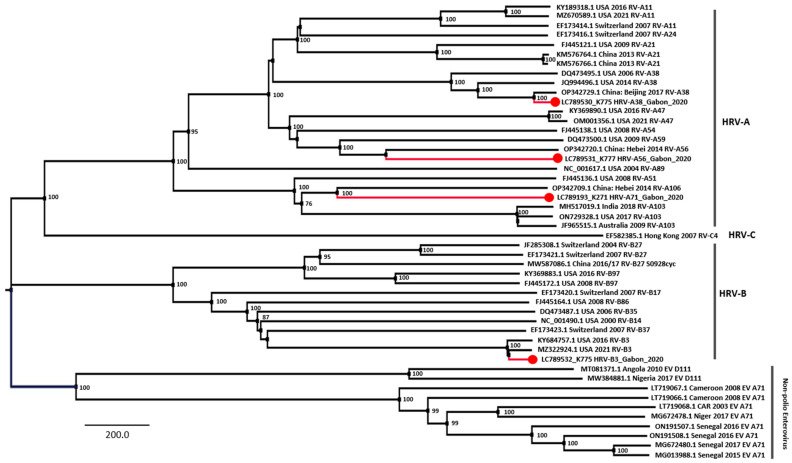
Phylogenetic tree of the whole genome of the human rhinovirus. Phylogenetic relationships were inferred using outgroup (Non-polio Enterovirus) rooting with the maximum likelihood tree with 1000 bootstrap replicates. Bootstrap values of greater than 70% are shown at nodes of the three. The full-length human rhinovirus genome sequence detected in Gabon is colored red and marked with a circle. The viral lineages are shown on the right side. Scale bar: Nucleotide substitutions per site.

Furthermore, the phylogenetic analysis of the whole-genome sequences was performed on three real-time PCR-positive samples, K271, K477, and K775, and complete or nearly complete genome sequences were obtained. Strains K271 and K477 belonged to HRV-A56 and -A71, respectively (Figure 3). The K775 sample included two different strains that belonged to HRV-A38 and HRV-B3, showing coinfection with two different HRV strains. The BLAST analysis of the two strains, HRV-A38 and -B3, detected in the K775 sample revealed that they shared the highest identity with the China-2016 (MW587063) and USA-2021 strains (OM001380), respectively.

### 3.6. Whole-Genome Sequencing of Gabonese hMPV Strain

**Figure 4:**
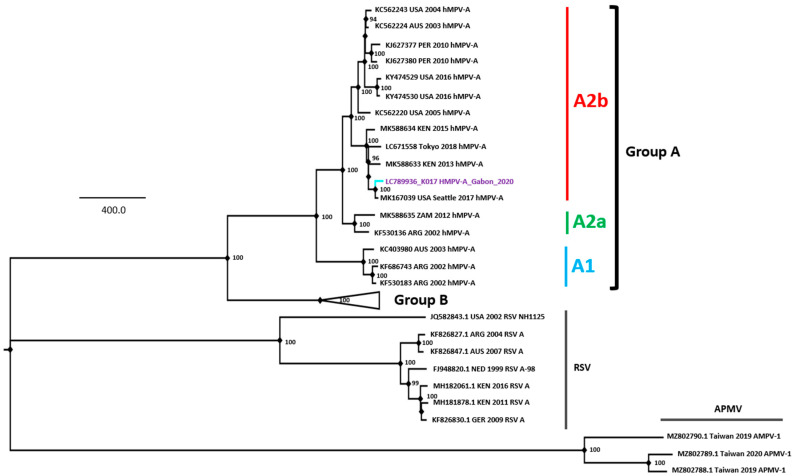
Phylogenetic tree of the whole genome of the human metapneumovirus. A maximum likelihood tree was inferred with 1000 bootstrap replicates. Bootstrap values of greater than 70% are shown at nodes of the three. Avian paramyxovirus (APMV) and RSV outgroup rooting were used for the reference, which are identified from the left to the right with the accession number, country, year, and species. The full-length human metapneumovirus genome sequence detected in the Gabon population is shown in blue. The viral lineages are shown on the right side. For better visualization of the sequence position, group B was collapsed and is shown as a triangle. Scale bar: Nucleotide substitutions per site.

In this study, we obtained the whole-genome sequence of hMPV (13,290 nucleotides) from sample K017, which showed the lowest Ct value when compared with that of the other two samples with high Ct values (37 and 40). To our knowledge, this is the first report on the sequence of hMPV in Gabon. To infer the phylogenetic relations of the hMPV detected in Gabon, a phylogenetic tree was constructed using a dataset of hMPV reference sequences (groups A and B) which was retrieved from the ViPR database (Figure 4). The Gabonese hMPV strain was classified into group A, particularly subgroup A2. In the BLAST analysis, the Seattle/USA/SC2938/2017 strain (MK167039) showed the highest homology to Gabonese hMPV-A2, with a nucleotide identity of 99.06%. Gabonese hMPV-A2 formed a cluster with Kenyan strains identified in 2013 and 2015, suggesting that this strain had been circulating on the African continent.

### 3.7. Whole-Genome Sequencing of Gabonese H3N2 IAV

Since no genomic sequence of IAV is available in Gabon, we attempted to determine the whole-genome sequences of the Gabonese IAV strains using two IAV-positive samples. We successfully obtained a whole-genome sequence from an IAV-positive sample, B001. Regarding the *HA* gene, the BLAST analysis showed that the most homologous isolates were H3N2 strains isolated in Connecticut, USA, in 2021 (99.54% identical to OP840967 and OP840959) (Table 4). The BLAST analysis of the NA nucleotide sequence showed the highest homology to the strain isolated in New York City, USA, in 2020 (99.50% identity to MW855529). The highest identity for each strain segment was observed in this study.

The BLAST analysis showed that PB2 had high homology with the strain isolated in Tennessee in 2020 (99.83% identical to MT467161), PA with the strain isolated in Georgia in 2019 (99.73% identical to MT168618), NS and PB1 with strains isolated in New York City in 2019 and 2022 (99.65% and 99.70% identical to MT056526 and ON531540, respectively), and NP and M with strains isolated in Connecticut in 2020 (99.55% and 99.90% identical to MT342019 and MT556994, respectively) (Table 4).

**Figure 5:**
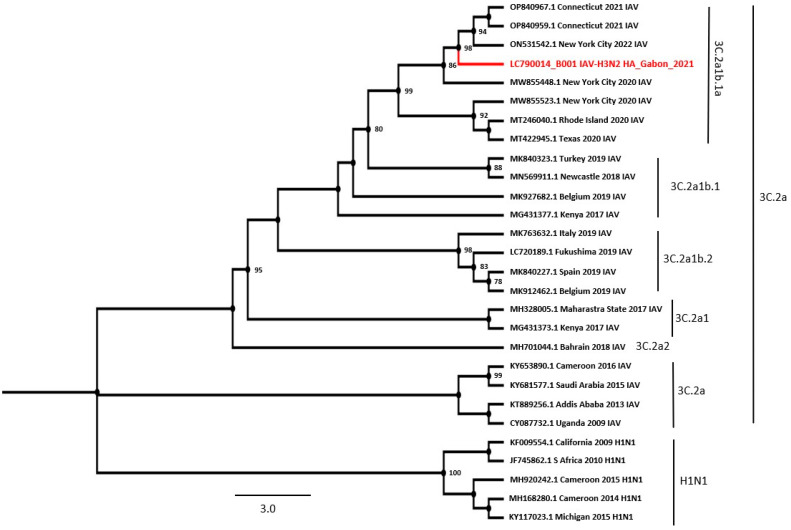
Phylogenetic tree of the whole coding region of HA. A maximum likelihood tree was inferred with 1000 bootstrap replicates, H1N1 was used as the outgroup to root the tree. Bootstrap values of ≥70% are shown at the nodes. The HA isolate in the present study clustered with the H3N2 subtype belonging to the USA lineage, clade 3C.2a, sub-clade 3C.2a1b.1a. The isolate used in this study is indicated in red. Scale bar: Nucleotide substitutions per site.

**Figure 6:**
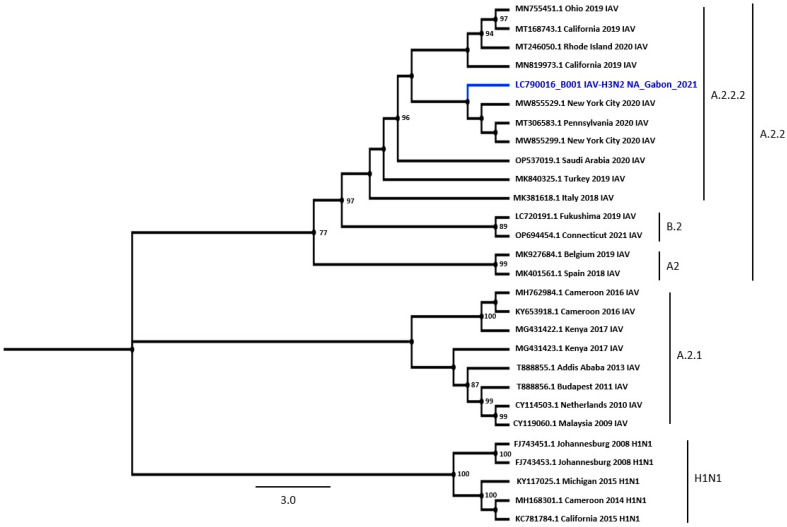
Phylogenetic tree of the whole coding region of NA. A maximum likelihood tree was inferred with 1000 bootstrap replicates, H1N1 was used as the outgroup to root the tree. Bootstrap values of ≥70% are shown at the nodes The NA isolate in the present study clustered with the H3N2 subtype belonging to the USA lineage, clade A.2.2, sub-clade A2.2.2. The isolates used in this study are shown in blue. Scale bar: Nucleotide substitutions per site.

To infer the phylogenetic relations between the *HA* and *NA* genes of Gabonese IAV, the reference strains were prepared using the NCBI database, including the highest BLAST homology sequences. As the IAV strain detected in this study was classified as H3N2, belonging to clade 3C.2a and 3C.2a1b.1a sub-clade, the reference sequences were limited to the H3 and N2 sequences (Figure 5 and Figure 6). Phylogenetic trees of the HA and NA sequences showed that the Gabonese strain was related to strains detected in the USA between 2020 and 2021, indicating that IAV had been imported from North America.

## 4. Discussion

In Gabon, although one study conducted in 2010–2011 reported epidemiological data for several respiratory viruses, such as PIVs, EV, RSV, IAV, and HRV [10], there are no genetic data available on respiratory viruses, possibly due to the lack of genetic surveillance studies conducted in the country until the COVID-19 pandemic. The current study revealed not only the circulation and distribution of respiratory viruses, but also the phylogenetic characteristics of the viruses. In this study, we found that 26% of nasal swab samples were positive for at least one respiratory virus targeted in this study. This prevalence was lower than that reported elsewhere in Africa [10,32]. It should be noted that this study involved mainly adults, unlike previous studies which focused on children aged <5 years, showing an advantage of this study in better understanding cases of respiratory virus infection in adults. The most common viruses detected in this study were EV (20.3%) and HRV (4.6%), while, in previous studies, the most common viruses were HAdV, EV, RSV, and HRV [10,33]. These differences between previous and current studies may reflect differences in study populations, especially in the participants’ age.

The onset of the COVID-19 pandemic has altered the circulation of ARIs worldwide, and, in many countries, COVID-19 has led to a decrease or increase in the positivity rate of several ARIs. Ren et al. showed that the positivity rate for influenza decreased considerably during the COVID-19 period when compared with that in the pre-COVID-19 period, whereas that for RSV increased during the same period [34]. In addition, Oh et al. showed that the appearance of COVID-19 was associated with an unprecedented and sustained decline in multiple respiratory viruses, among which HRV was the only agent that resurged to levels equal to those of previous years [5]. HRV infections were first observed in children after schools reopened, in contrast to other non-enveloped viral infections, which were suppressed after schools reopened. The current study shows a similar trend in Gabon, with certain respiratory viruses that were predominant before COVID-19 (such as HAdV and RSV) declining considerably during COVID-19. However, the detection rate of EV in this study was high, similar to that reported in 2014 in Gabon, possibly indicating that Gabonese people maintained a similar lifestyle during the pandemic to that before COVID-19; that is, the majority of the Gabonese population lived in a large family that could contribute to easy EV transmission. In addition, although EV was detected in all cities, some viruses, including IAV, were only detected in certain cities, indicating a regional bias in virus circulation in Gabon. This result corroborates the trend of a previous study conducted during 2010–2011, before COVID-19 in Gabon [10]. However, further investigation is required to validate the effects of the COVID-19 pandemic on the incidence of other respiratory viral diseases.

This is the first study to report the molecular characterization of HRV, hMPV, and IAV strains, including the whole-genome sequences of hMPV and IAV.

The genotyping analysis of HRV suggested that all HRV genotypes (A, B, and C) circulated in Gabon, and a similar situation was reported in 2021 in Cameroon [31], a neighboring country of Gabon. In Africa, HRV-A and HRV-C strains generally predominate over HRV-B strains [35]. This trend was confirmed in the present study, in which HRV-A (51.2%) was predominant, followed by HRV-B and HRV-C (24.4%). BLAST analyses of HRV genome sequences inferred that the Gabonese HRV strains originated from the USA (six strains), Cameroon (two), China (three), Australia (one), and Nepal (one), indicating high diversity in the origins of HRV imported to Gabon. In particular, we found a case of HRV coinfection with two different strains (HRV-A38 and HRV-B3) (Figure 2). Both HRV types were minor in this study; however, further surveillance is required to identify regions at risk of HRV coinfections.

HRV has three species: A–C. Gabonese strains were distributed across all species, although genotype A was the most predominant. To date, the data on the association between clinical severity and HRV species in children are contradictory. Several studies have suggested no link or similarity between HRV species and clinical presentation [36], whereas others have suggested a potential association between the disease severity and HRV-C or other virus types [36]. For example, Bruning et al. showed that the distribution of HRV species in children with respiratory distress who required admission to an intensive care unit did not differ significantly from that in non-hospitalized children [37]; further clinical data are required to clarify the situation in Gabon. Conversely, more recent studies have shown that severe illnesses, such as low oxygen saturation, cough, and wheezing, are more common in patients infected with HRV-C than in those infected with HRV-A or HRV-B [38]. Although many participants in this study were adults, researchers and physicians should carefully monitor the severity of HRV infections in Gabon.

hMPV genome data from Africa are sparse, and information on genome wide diversity is limited [39]. The Gabonese hMPV strains detected in this study were classified into groups A and A2, as observed in several African countries [40]. In the present study, the whole-genome sequence of an hMPV strain from Gabon was determined for the first time and compared with genomes previously published worldwide. According to the BLAST analysis, this strain was genetically close to the USA strain (Seattle/USA/SC2938/2017). hMPV-A2 was the most frequently observed subgroup and was further divided into two proposed sub-lineages (A2a and A2b). In the present study, the sub-lineage of the Gabonese strain of hMPV was designated as A2b. It was reported that hMPV group A infection in children caused a more severe illness than that caused by group B infection [41], whereas several studies reported that hMPV groups and viral load showed little impact on the symptom severity in adults with ARI [42]. With this in mind, the detection of this strain in our results is an important first step in establishing a system to control and prevent hMPV infections in children in Gabon.

This is the first report to analyze the genomic sequence of IAV identified in Gabon, with the successful sequencing of all segments of an IAV strain. The analysis of the *HA* and *NA* genes showed that the strain detected in this study was classified as an H3N2 subtype, clade 3C.2a and sub-clade 3C2a1b.1a. Regarding the *HA* gene, the most homologous isolates were H3N2 strains from Connecticut, USA, whereas the *NA* gene showed the highest homology with the strain from New York City. According to the HA and NA phylogenetic trees, the Gabonese strain belonged to the same cluster as the USA vaccine strain 2020–2021 (3C2A–3C3A), including two subgroups (2a.1 and 2a.2), as observed in several African countries [43,44,45,46,47]. Therefore, our study represents the first major advance in our knowledge of IAV circulating in Gabon.

The limitations of the present study include the variability of the sample numbers in each city and the lack of clinical data, reflecting the properties of samples that had been originally collected for regular COVID-19 testing. These limitations make it difficult to identify the risk factors for ARI infections in Gabon. From the perspective of establishing the next surveillance program, it may be necessary to collect clinical and demographic data simultaneously in the hospital. In addition, the number of samples should be set to become proportional to the population in each region, enabling us to accurately understand the prevalence of respiratory viral infections, and some viruses, including Bocaviruses, should be included.

## 5. Conclusions

This study revealed the genetic characteristics of the respiratory viruses that appeared in Gabon during the COVID-19 pandemic. For the first time, hMPV was detected in Gabon, and the genetic diversity of HRV, hMPV, and IAV was verified. This information on the respiratory virus strains circulating in Gabon is important for future studies and the establishment of a surveillance system in the country. Further investigation is required to validate the effect of the COVID-19 pandemic on the incidence of other respiratory viral diseases, and to develop an effective prevention strategy for ARIs in Gabon.

## Figures and Tables

**Table 1:** Detection of respiratory viruses of public health concern in Gabon.

Variable	Characteristics	N (%)	P (%)	EV (%)	HRV (%)	OC43 (%)	HAdV (%)	hMPV (%)	IAV (%)	PIV (%)
Overall prevalence	Samples	582	154 (26.5%)	118 (20.3%)	27 (4.6%)	7 (1.2%)	5 (0.9%)	3 (0.5%)	2 (0.3%)	3 (0.5%)
Sex	Women	268 (46.1)	72 (26.9%)	59 (22%)	9 (3.4%)	5 (1.9%)	0	1 (0.4%)	0	1 (0.4%)
	Men	247 (42.4)	65 (26.3%)	47 (19%)	15 (6.1%)	1 (0.4%)	4 (1.6%)	1 (0.4%)	2 (0.8%)	2 (0.8%)
	unspecified	67 (11.5)	17 (25.4%)	12 (17.9%)	3 (4.5%)	1 (1.5%)	1 (1.5%)	1 (1.5%)	0	0
Age	0–5 years	15 (2.6)	9 (60%)	3 (20%)	5 (33.3%)	0	0	1 (6.7%)	0	1 (6.7%)
	6–17 years	35 (6.1)	10 (28.6%)	4 (11.4%)	4 (11.4%)	0	2 (5.7%)	0	1 (2.9%)	1 (2.9%)
	18–60 years	452 (77.7)	112 (24.8%)	92 (20.4%)	16 (3.5%)	7 (1.5%)	1 (0.2%)	1 (0.2%)	1 (0.2%)	1 (0.2%)
	>60 years	20 (3.4)	5 (25%)	5 (25%)	0	0	0	0	0	0
	unspecified	60 (10.2)	18 (30%)	14 (23.3%)	3 (5%)	0	1 (1.7%)	1 (1.7%)	0	0
Region	Bitam	48 (8.3)	15 (31.2% [19;46])	14 (29.2%)	1 (2.1%)	0	0	0	0	0
	Lambaréné	82 (14.1)	24 (29.3%)	17 (20.7%)	7 (8.5%)	0	3 (3.7%)	0	2 (2.4%)	1 (1.2%)
	Libreville	410 (70.4)	106 (25.9%)	79 (19.3%)	16 (3.9%)	7 (1.7%)	1 (0.2%)	3 (0.7%)	0	2 (0.5%)
	Makokou	5 (0.9)	1	1	1	0	0	0	0	0
	Mouila	14 (2.4)	3	3	0	0	0	0	0	0
	Tchibanga	9 (1.6)	5	4	1	0	0	0	0	0
	unspecified	14 (2.4)	0	0	0	0	0	0	0	0

EV, enterovirus; HRV, human rhinovirus; OC43, coronavirus OC43; HAdV, human adenovirus; hMPV, human metapneumovirus; IAV, influenza A virus; PIV, parainfluenza virus; CI, confidence interval; N, sample number; P, positive number.

**Table 2:** Detection of respiratory viruses per month during the study.

Collection Year	Collection Month	Sample Number	Detected Virus	CT Values *	Number	Total
2020	March	43	EV		8	14 (33%)
			HAdV/hMPV *	32.72/36.84	1	
			hMPV		1	
			HRV		4	
	April	349	EV		64	88 (25%)
			HAdV		2	
			HAdV/PIV4a *	39.19/39.42	1	
			HRV		10	
			EV/HRV *	37.3/38.28 and 37.7/39.5	2	
			OC43		7	
			PIV 4a		1	
			PIV3		1	
	May	52	EV		8	11 (21%)
			EV/HRV *	25.98/38.63	1	
			HRV		2	
	June	24	EV		8	8 (33%)
	July	23	EV		8	9 (39%)
			HRV		1	
	August	7	EV		1	2 (29%)
			EV/HRV *	33.51/39.88	1	
	September	6	EV		2	3 (50%)
			HRV		1	
	October	3	-		0	0
	November	19	EV		9	12 (63%)
			hMPV		1	
			HRV		1	
			EV/HRV *	27.72/36.63	1	
	December	1	-		0	0
2021	January	17	-		0	0
	February	12	-		0	0
	March	10	-		0	0
	April	4	-		0	0
	May	NC	-		-	-
	June	NC	-		-	-
	July	12	EV		1	7 (58%)
			HRV		1	
			EV/HRV *	26.52/37.36, 28.07/38.45 and 21.98/37.71	3	
			IAV		1	
			IAV/EV *	34.85/38.86	1	

EV, enterovirus; HRV, human rhinovirus; IAV, influenza A virus; PIV, parainfluenza virus; OC43, coronavirus OC43; HAdV, human adenovirus; hMPV, human metapneumovirus. (*) indicates cases of coinfection, (-) indicates no detection, and (NC) indicates no sample collection.

**Table 3:** Genotyping of human rhinovirus (HRV) based on the VP4/VP2 region.

	HRV-A	HRV-B	HRV-C	Total
Region	N	N	N	N
VP4/VP2	8	4	3	15

**Table 4:** List of the influenza A virus (IAV) strains which showed the highest identity to each segment of the IAV B001 strain of the current study.

Segment	Genes	Highest Homologous Influenza Virus	GenBank Accession Number	Percentage of Homology
1	*PB2*	A/Tennessee/26/2020 (H3N2)	MT467161	99.83%
2	*PB1*	A/New York City/PV42662/2022 (H3N2)	ON531540	99.70%
3	*PA*	A/Georgia/44/2019 (H3N2)	MT168618	99.73%
4	*HA*	A/Connecticut/09/2021 (H3N2)	OP840967	99.54%
5	*NP*	A/Connecticut/09/2020 (H3N2)	MT342019	99.55%
6	*NA*	A/New York City/PV08762/2020 (H3N2)	MW855529	99.50%
7	*M*	A/Connecticut/21/2020 (H3N2)	MT556994	99.90%
8	*NS*	A/New York/61/2019 (H3N2)	MT056526	99.65%

## Data Availability

The original contributions presented in the study are included in the article, further inquiries can be directed to the corresponding author.
